# Age Matters: Objective Gait Assessment in Early Parkinson's Disease Using an RGB-D Camera

**DOI:** 10.1155/2019/5050182

**Published:** 2019-06-13

**Authors:** Beatriz Muñoz Ospina, Jaime Andrés Valderrama Chaparro, Juan David Arango Paredes, Yor Jaggy Castaño Pino, Andrés Navarro, Jorge Luis Orozco

**Affiliations:** ^1^Fundación Valle del Lili, Departamento de Neurología, Cra 98 #18-49, Cali 760032, Colombia; ^2^i2t Research Group, Universidad Icesi, Cl. 18 #122-135, Cali, Colombia; ^3^Centro de Investigaciones Clínicas (CIC), Fundación Valle del Lili, Cra 98 #18-49, Cali 760032, Colombia; ^4^Estudiante de Doctorado en Ciencias Biomédicas, Universidad del Valle, Cra. 4b #36-00, Cali 760043, Colombia

## Abstract

**Background:**

Gait alterations are hallmarks for the diagnosis and follow-up of patients with Parkinson's disease (PD). In normal conditions, age could affect gait dynamics. Although it is known that objective assessment of gait is a valuable tool for diagnosis and follow-up of patients with PD, only few studies evaluate the effect of aging on the gait pattern of patients with PD.

**Objective:**

The purpose of this study was to assess differences in gait dynamics between PD patients and healthy subjects and to investigate the effects of aging on these differences using a low-cost RGB-D depth-sensing camera.

**Methods:**

30 PD patients and 30 age-matched controls were recruited. Descriptive analysis was used for clinical variables, and Spearman's rank correlation was used to correlate age and gait variables. The sample was distributed in age groups; then, Mann–Whitney *U* test was used for comparison of gait variables between groups.

**Results:**

PD patients exhibited prolonged swing (*p*=0.002) and stance times (*p* < 0.001) and lower speed values (*p* < 0.001) compared to controls. This was consistent in all age groups, except for the one between 76 and 88 years old, in which the controls were slower and had longer swing and stance times. These results were statically significant for the group from 60 to 66 years.

**Conclusion:**

Gait speed, swing, and stance times are useful for differentiating PD patients from controls. Quantitative gait parameters measured by an RGB-D camera can complement clinical assessment of PD patients. The analysis of these spatiotemporal variables should consider the age of the subject.

## 1. Introduction

PD is the second most common neurodegenerative disorder worldwide, and its incidence is highly increasing even surpassing other neurological diseases such as Alzheimer's disease. Primary motor symptoms of PD include bradykinesia, rigidity, postural instability, and tremor [1]. Some of these symptoms affect the lower limbs and alter gait pattern of patients.

Spatiotemporal characteristics of gait are recognized as valuable tools for evaluation and decision-making processes regarding treatment of several illnesses, such as Parkinson's disease (PD), stroke, and multiple sclerosis [2]. Shortened steps, reduced travel speed, increased support phase, and reduced swing phase are some of gait changes reported in PD patients.

Usually, gait is examined via visual assessment (naked eye) by trained physicians or neurologists. Although this approach is informative, the results from these observations are often limited because they depend on the restrictive consultation time and the experience of the clinician who performed the assessment [3, 4]. In this context, gait analysis through naked eye becomes even more complex if we consider that about 35% of adults over 70 years have gait changes [5] including slower and shorter steps [6, 7]. This means that even healthy elderly patients may have gait changes similar to those found in PD. Little is known about the relationship between gait and age in patients with PD [8–10], and most studies compare spatiotemporal gait variables without considering the age as a possible confounder factor.

Technology supporting human motion analysis has made important advances in the past three decades; however, despite being useful, the routine applicability and accessibility of this technology have been limited [11]. Gait parameters can easily be obtained using three-dimensional motion analysis cameras, foot switches, body-mounted inertial tracking unit sensors, instrumented walkway systems (e.g., GAITRite), and accelerometers [3]. These instruments can provide accurate quantitative data regarding many variables; however, their routine implementation in clinical environments requires a high-quality patient preparation, longer time, expensive equipment, accessibility, and technical expertise and demands a special place [12–14].

Portable motion sensing devices, such as the Microsoft Kinect®, are depth cameras originally developed for video gaming. This technology uses infrared light to detect anatomical landmark positions in three dimensions, allowing them to analyze gait and limb movements [15]. This device has been proposed as a solution to the constraints of objective assessment of gait analysis because of its portability, low cost, convenience, and simple use in clinical and research laboratories [3]. Several clinical studies have favored the use of Kinect®, reporting adequate concordance with motion and gait laboratories on the assessment of healthy subjects' identification of steps [16], postural control, speed, length of step, and gait cycle [13] and the assessment of movements of upper extremities [17]. However, there is still a paucity of research regarding potential usefulness of the Kinect™ system for assessing gait in clinical populations.

The aim of this research is to perform a quantitative gait analysis using a portable movement capture system (Kinect) to describe the relationship between age and gait variables in PD patients and to compare gait changes between PD patients and healthy subjects according to age distribution.

## 2. Methods

### 2.1. Patient Selection and Clinical Assessment

Thirty PD patients and 30 healthy subjects (age-matched) were recruited for this cohort study. PD diagnosis was made by the movement disorder specialist at the institution following the UK Parkinson's Disease Society Brain Bank diagnostic criteria [18]. Exclusion criteria considered the absence of any other neurological disease or severe comorbidity, which may affect gait, the absence of dementia, and the ability to walk without aids. All participants were evaluated in a single session by an expert neurologist who administered the MDS-UPDRS part III to determine the severity of motor symptom. The Dynamic Gait Index (DGI) and the Freezing of Gait Questionnaire (FOGQ) were also administered by the neurologist. Classically, patients with greater motor involvement have higher scores in the MDS-UPDRS part III, higher scores in FOGQ, and lower scores in DGI. Montreal Cognitive Assessment (MoCA) test was administered as a cognitive screening tool. Data on PD characteristics were also obtained for the PD group. Institutional review boards of both the Universidad Icesi and Fundación Valle del Lili, Cali, Colombia, approved the study. This work was conducted according to the Helsinki Declaration. Informed consent was obtained from all subjects (patients and controls).

### 2.2. Gait Analysis Method: E-Motion Capture System and Kinect Sensor

**Figure 1:**
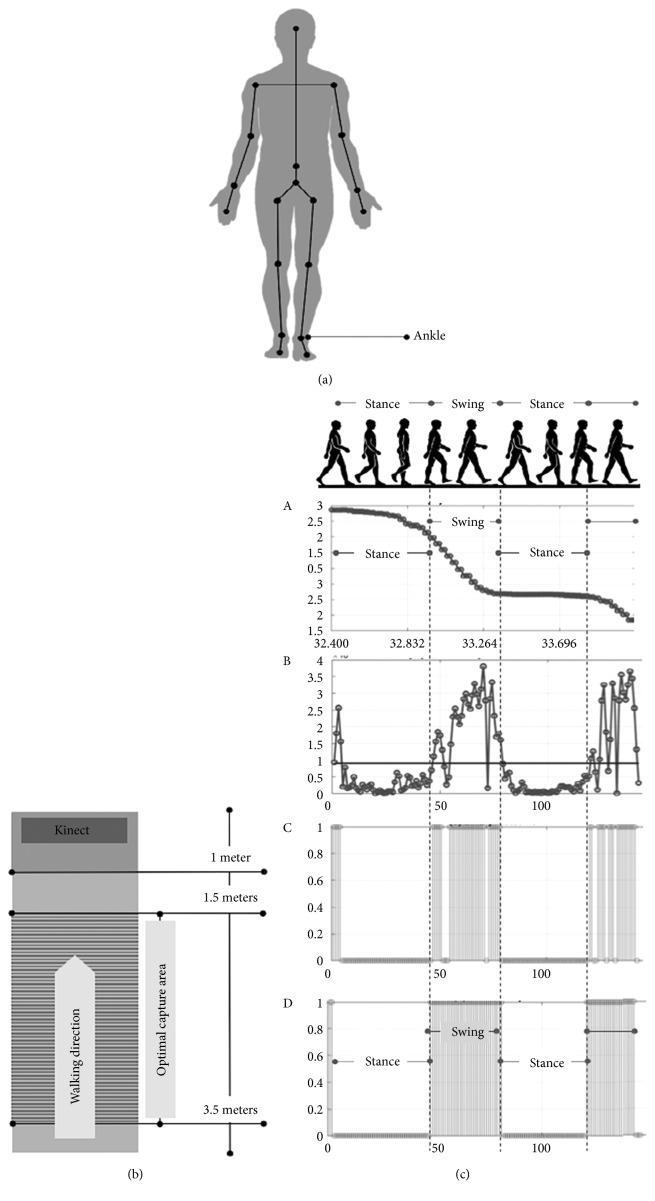
(a) General setting and results obtained with e-motion system. (b) Capture area. (c) Recorded and binarized signal.

The Microsoft Kinect sensor has an RGB-D camera designed for applications in the gaming industry. Kinect is able to detect and track 20 different body joints (Figure 1(a)). Comparisons between the Kinect and benchmark references have shown a high agreement [19, 20]. Also, this device has been used in different research areas, like e-health [21, 22], security and surveillance [23–25], and UAV and robot vision [26]. In e-health approaches, this device has displayed good reliability in clinical context [27]. Furthermore, Kinect has been tested for PD diagnosis; some researchers have used this device to measure and quantify different symptoms like gait [21], arm swing [28], postural instability, and tremor [29] in PD patients.

Therefore, we used the e-motion capture system, which contains the e-motion software developed by the CENIT research center from Universidad Icesi. This system contains a motion sensing device (Kinect™ V1 or V2), a computer with the e-motion software, free interface capture area, and a rater (physician or trained nurse). The e-motion software captures [19] skeleton information from the Kinect and records it in the computer, using an ID to identify the patient in later analysis. From the skeleton information, we can extract information from different joints and analyze it. For the patients' ankles, we obtain a set of coordinate points with distance (vertical axis) and time (horizontal axis) information (Figure 1(c)).

Using the e-motion software, we extract the ankle information from the captured skeleton information and postprocess it to obtain gait parameters. In this postprocessing, we use wavelet transform to convert the distance versus time information into a binary signal with swing and stance phases differentiated. This binary signal allows us to compute gait parameters, such as swing time, stance time, and speed used for gait analysis. Although it is possible to obtain additional parameters, like stride length, only relevant parameters are used in a clinical context.

To obtain gait information, 30 PD patients and 30 controls were recruited. For this study, the subjects were instructed to walk on a flat walkway (approximately 4 meters in length and 2.5 meters wide) toward the Kinect® device (Figure 1(b)). For each subject, we performed three barefoot walking trials; for which all PD patients were evaluated in the “ON” state. For this study, the acceptable field of view was restricted to a range of 1.5–3.5 m from Kinect™. This distance allowed for a minimum of one full gait cycle per limb to be recorded per walking trial.

Recently, Kinect has gained popularity for various applications in PD diagnosis. However, in this research, Kinect limits the capture area, which restricts the walking length to 4 meters. Some researchers have addressed this limitation using multiple Kinect devices [12], but synchronizing these devices is challenging. Main advantages of the Kinect as a sensor for PD diagnosis are portability, affordability, and touchless.

### 2.3. Signal Processing Techniques and Gait Phase Estimation

Wavelets have demonstrated their utility in biomedical signal analysis since 1996, when Michael Unser suggested some applications for wavelet techniques on biomedical applications like noise reduction, image enhancement, and detection of microcalcifications in mammograms; image reconstruction and acquisition schemes in tomographies and magnetic resonance imaging; and multiresolution methods for registration and statistical analysis of functional images of the brain. As a conclusion, Unser claims that wavelet transforms are not a panacea and should be used with caution [30]. Additionally, wavelets are now being applied in gait phase extraction, biomedical signal compression [31], recognition of cardiac patterns [32], EMG classification and decoding [31, 33], main features detection and extraction on ECG [32] and PPG [34], and diagnosis of epilepsy [35].

For the gait phase identification, we apply wavelet decomposition using the Daubechies family in one-level decomposition, with eight vanishing moments (db8) because in previous research, it was one of the decomposition with less average error [36]. In one-level decomposition, we obtain two resultant signals, one with approximation coefficients or in this case a gait signal denoised, and the second one generates a signal with detailed coefficients, which reflects clear changes in gait phases. This decomposition allows us to obtain information in two domains: spectral and time. The swing phase corresponds to the moment where the ankle is in motion and the stance phase to the moments where the ankle is static on the floor. After the wavelet decomposition, we establish the mean value as a threshold (denoted by horizontal line in Figure 1(c)) to define swing and stance phases. The swing phase is defined as the values above the average value and the stance phase is defined as the values below the average value. This classification was based on the signal structure. The structure suggests that moments with descending changes represent a swing phase and the other ones represent the stance phase. We use the coefficients from the second signal of the one-level decomposition to generate a binary signal, in which the one values represent the swing phase and the zero values represent the stance phase (Figure 1(c)). Using binary signal, we established a time and distance reference in each phase. Based on these values and on the ankle information, we estimate the following variables:

#### 2.3.1. Stance Time

It is the duration of time (s) of limb movements tracked in the support phase during a walking trial in the acceptable field of view of Kinect.

#### 2.3.2. Swing Time

It is the duration of time (s) of limb movements tracked in the swing phase during a walking trial in the acceptable field of view of Kinect.

#### 2.3.3. Speed

It is the rate of motion, measured in meters per second (m/s), during a walking trial in the acceptable field of view of Kinect.

### 2.4. Statistical Methods and Data Analysis

Categorical variables were expressed with relative frequencies and total counts. Continuous variables were assessed with median and interquartile range or with mean and standard deviation based on their normality distribution determined by the Shapiro–Wilk test. A bivariate analysis comparing PD patients and healthy subjects was based on Mann–Whitney U and Pearson's *X*^2^ test. Spatiotemporal gait variables of each leg were analyzed together, independently of their laterality. To assess gait-related changes (speed, stance time, and swing time) with respect to age, Spearman's rank correlation was used. Subsequently, groups were classified according to the age quartiles distribution and bivariate analyses were made for each age group. A significant difference was reached with *p* values ≤ 0.05. Statistical analyses were performed using STATA© 13.0 (StataCorp, TX USA).

## 3. Results

Sixty subjects (30 PD patients and 30 healthy subjects) were included. Both groups had a median age of 66 years (IQR 59–75). No significant differences were found by comparing the groups for sex, age, or MoCA test score.  Table 1 shows the sociodemographic characteristics of the sample.

### 3.1. Clinical Background and Parkinson's Disease Characteristics

The median duration of the disease was 5 years (IQR 1–7). Hoehn and Yahr stage classification was stage I for 17% of the PD patients, stage II for 73%, and stage III for the remaining 10%. The mean MDS-UPDRS part III score was 39.06 (±13.74), the mean DGI was 19.73 (±4.07), and the mean FOGQ score was 6.73 (±4.95).

When PD clinical characteristics were classified according to age distribution, compared with the other age groups, the patients between 76 and 88 years displayed the highest MDS-UPDRS part III 43.5 (±8.84), the highest FOGQ (7.83 ± 4.95), and the lowest DGI (18.83 ± 6.27) scores. Contrarily, patients between 67 and 75 years displayed the lowest MDS-UPDR part III (33.66 ± 12.44) scores and the ones between 40 and 59 years the lowest FOGQ (4.87 ± 5.59) and the highest DGI (21.62 ± 2.87).  Table 2 shows the PD characteristics for each patient group according to the age distribution.

### 3.2. Gait Differences between Groups

Compared to the control group, PD patients showed prolonged swing times (PD = 0.90, healthy = 0.81 seconds, *p*=0.002), prolonged stance times (PD = 1.29, healthy = 1.16 seconds, *p* < 0.001), and lower speed values (PD = 0.86, healthy = 0.94 m/s, *p* < 0.001).  Table 3 shows the comparison of gait parameters measured using the e-motion capture system.

### 3.3. Gait-Related Changes with respect to Age

When gait variables and age were related, a negative correlation was found for speed (PD: rho = −0.072, healthy: rho = −0.360) and positive correlations were found for swing (PD: rho = 0.086, healthy: rho = 0.40) and stance times (PD: rho = 0.07, healthy: rho = 0.27). These correlations were significant only in the healthy subjects group (speed, *p*=0.004; stance time,  *p*=0.035; swing time, *p*=0.001).

Below 76 years, compared to healthy subjects, PD patients exhibited lower speed values and prolonged swing and stance times. These results were statistically significant for the 60 to 66 years group and almost achieved significance in the one between 67 and 75 years. Over 75 years, healthy subjects displayed lower speed values and prolonged swing and stance times compared to PD patients; these differences were no statically significant (see Table 3).

## 4. Discussion

Gait assessment is fundamental for the diagnosis and follow-up of patients with PD. Since the evaluation of motor alterations can be highly subjective and taking into account that the use of technologies for gait analysis is expensive and is almost restricted for research purposes, we attempted to assess the main gait variables using a low-cost system that can be easily accessed during a medical consultation. According to our results, compared with healthy subjects, PD patients' gait is slower and has longer swing and stance times. While this is true, these changes are highly influenced by the patient's age and disease stage.

### 4.1. PD Patients Are Slower and Had Prolonged Swing and Stance Times

As expected, based on existing research, we found lower speed values in the PD group. This could be explained by bradykinesia and gait changes related to the disease, such as high cycle time, a high step number, and a shortened stride length, all of which are related to a slow gait [37, 38].

Regarding differences in swing time, higher values were found in the PD group, which was unexpected based on the results proposed by previous studies [38, 39]. We think this could be explained by the fact that PD patients are slower and need more time to perform a step. This means that both swing and stance phases are prolonged. Compared to healthy subjects, the stance time values were prolonged in the PD group. Previous studies on gait analysis in PD have also shown a higher stance time phase compared to controls, which they have associated with longer double limb support [19, 22].

### 4.2. Speed, Stance, and Swing Time Differences Are Influenced by Age

When the sample was age-stratified, we observe gait differences change depending on the age of the compared groups. This finding can be explained by two factors: the first one is associated with the progression and the burden of the disease and the second one is related to the gait changes induced by the aging process in the control group.

Nonsignificant differences in the younger group: although descriptive results showed that patients in the younger group were slower and had prolonged swing and stance times compared to controls, these results did not reach significance. Patients in the younger group had the shortest disease duration (1 IQR 0–4), the second lowest MDS-UPDRS part III, the highest DGI, and the lowest FOGQ score which could be associated with a lower disease burden and fewer gait changes. Therefore, differences in gait kinematics in young PD patients can be very subtle, especially, in patients in early disease stages, in which lower limb involvement is less frequent and gait alterations are almost restricted to arm swing changes.

Statistically significant results (*p* < 0.001) were found for speed in the 60 to 75 years group; this finding supports that speed changes could be useful in the differentiation between PD patients and healthy subjects in that age range. Swing and stance time differences were only significant between 60 and 66 years (*p* < 0.001) and showed a trend to reach significance (*p*=0.05) in the 67 to 75 years group, which could be associated with the sample size increasing type 2 error.

Nonsignificant differences in the oldest group: although patients in this group have the highest burden of disease (highest scores in the MDS-UPDRS and FOGQ and the lowest scores in the DGI), healthy subjects in this group already have gait changes induced by age. As will be discussed later, older subjects tend to be slower and their gait kinematic is also altered in relation to the physiological aging process.

### 4.3. Gait Changes Related to Age Are Different between PD Patients and Healthy Subjects

For the healthy group, a significant negative correlation was found between age and speed; this finding is similar to the reports in elderly Caucasian and Asian populations [40–42]. The physiologic loss of muscle strength, the deterioration of motor cortical regions, and the development of a more cautious with slower speed and a reduced stride length [43] could explain why gait slowness is negatively correlated with age. Although there are no studies that correlate the swing or stance times with aging, our results suggest that there is a positive relationship between age and both gait variables. Reductions in stride length [44], reductions in walking speed, and reductions in cadence [45], which are associated with a longer stance time and prolonged double support times in the elderly population, could explain this finding.

For the PD group, the correlations between age and the gait spatiotemporal variables mentioned above were not significant. PD patients have different patterns of motor impairment, and the progression of motor symptoms varies according to the age of onset and the duration of the disease. Some studies suggest that patients with an older age of onset have a faster rate of motor progression, worsening of motor symptoms in a shorter time, and greater balance impairment than those with early onset of disease [46, 47]. This individual variability in the progression of PD could explain why the correlations between the age of the patients and the spatiotemporal variables of gait were not significant.

### 4.4. Limitations and Advantages

The data obtained from the other Kinect reference points were not considered because the main objective of this work was to characterize gait only using the data on lower limbs. Space-related variables (e.g., asymmetry) were not calculated because the test field captured by Kinect® was not long enough to estimate them. However, the use of Kinect® in this clinical context has reported relative and overall reliability regarding spatiotemporal parameters [21, 48, 49] further advances in software and hardware are essential to enhance Kinect's sensitivity for kinematic measurements [50, 51]. Nevertheless, because Kinect is an inexpensive and portable device, it provides opportunities in the field of medicine and telemedicine, allowing easy access to gait assessment in clinical space and allowing remote diagnose in rural areas, where there are no clinical experts.

### 4.5. Challenges and Future Research

Precision medicine is a growing field that enables objective characterization of patients. E-motion is a diagnostic aid that could be used with other complementary technologies to improve and quantify gait assessment of patients diagnosed with neurological diseases such as PD. We consider that the strategy used for data collection presents relevant advantages in terms of cost, accessibility, and space [21, 48], compared to gait laboratories. Although Kinect system is no longer in production, there are other RGB-D cameras that can be used with the e-motion software. The operation of these cameras does not require specialized training, and they can be placed in almost any doctor's office without making major adjustments to the test area, making the device adaptable to any medical environment. In future research, a larger number of subjects will be evaluated for establishing cutoff points that could help in the differentiation of patients diagnosed with PD from controls and to monitor the symptoms and severity of the disease. The analysis of the information obtained from upper limbs and technical limitations of our approach will be considered in the development of future research.

## 5. Conclusion

The development and improvement of new and more portable technologies may allow for an objective evaluation of quantitative gait parameters that can complement clinical assessment and follow-up of patients, potentially detecting earlier stages of neurodegenerative diseases such as PD. Age is an important factor that affects gait; therefore, the analysis of spatiotemporal variables should be individualized, considering the age of the patient.

## Figures and Tables

**Table 1:** Clinical background and characteristics of the sample.

Variables	PD patients	Healthy subjects	*p* value
	(*n* = 30)	(*n* = 30)	
Age			
Years (median, IQR)	66 (IQR 59–75)	66 (IQR 59–75)	0.88
40–59	8 (26.6%)	8 (27)	0.90
60–66	8 (26.6%)	7 (25%)	
67–75	8 (26.6%)	9 (28%)	
76–88	6 (20%)	6 (20%)	

Gender			
Male	17 (57%)	19 (63%)	0.60
Female	13 (43%)	11 (36%)	

Education			
Elementary school	9 (30%)	5 (17%)	0.20
Highschool	10 (33%)	10 (33%)	
Graduate	11 (37%)	15 (50%)	

Occupation			
Employee	8 (27%)	15 (50%)	0.08
Housewife	7 (23%)	5 (17%)	
Retired	15 (50%)	10 (33%)	
MoCA test	22 (IQR 16–26)	22.5 (IQR 21–24)	0.57

**Table 2:** PD patient characteristics by age group.

Variables	40–59 years	60–66 years	67–75 years	76–88 years
	(*n* = 8)	(*n* = 7)	(*n* = 9)	(*n* = 6)
Years of disease	1 (IQR 0–4)	6 (IQR 3–7)	6 (IQR 2–7)	5.5 (IQR 1–9)

Age at diagnosis	52 (43–56.5)	59 (55–63)	64 (61–70)	74.5 (71–76)

Subtype of PD				
TD	3 (37.50%)	1 (14.29%)	2 (22.22%)	2 (33.33%)
PIGD	5 (62.50%)	6 (85.71%)	7 (77.78%)	4 (66.67%)

Hoehn and Yahr scale				
I	4 (50%)	0 (0%)	1 (11.11%)	0 (0%)
II	3 (37.50%)	6 (85.71%)	7 (77.78%)	6 (100%)
III	1 (12.50%)	1 (14.29%)	1 (11.11%)	0 (0%)

Test				
MDS-UPDRS part III	39.5 ± 18.44	41.71 ± 13.12	33.66 ± 12.44	43.5 ± 8.84
FOGQ	4.87 ± 5.59	7.71 ± 4.99	6.88 ± 4.72	7.83 ± 4.95
DGI	21.62 ± 2.87	19.42 ± 3.15	18.88 ± 3.98	18.83 ± 6.27
Patients with fall risk	1 (6.25%)	1 (6.67%)	4 (23.53%)	2 (16.67%)
MoCA test	23 (20.5–24)	24 (24–26)	20 (15–24)	18.5 (17–22)

**Table 3:** Spatiotemporal gait parameters obtained from the e-motion capture system in the PD patient group and the healthy subjects group.

Gait variable	Speed (m/s)			Swing time (s)			Stance time (s)		
Age/group	PD patients	Healthy subjects	*p* value	PD patients	Healthy subjects	*p* value	PD patients	Healthy subjects	*p* value
All ages (*n* = 60)	0.86 (IQR 0.73–0.93)	0.94 (IQR 0.86–1.14)	**<0.001**	0.90 (IQR 0.80–1.09)	0.81 (IQR 0.71–0.92)	**0.002**	1.29 (IQR 1.13–1.57)	1.16 (IQR 0.95–1.27)	**<0.001**

40 to 59 years (*n* = 16)	0.89 (IQR 0.80–1.04)	0.97 (IQR 0.89–1.12)	0.10	0.86 (IQR 0.71–0.94)	0.77 (IQR 0.70–0.84)	0.19	1.22 (IQR 1.07–1.40)	1.1 (IQR 0.99–1.27)	0.13

60 to 66 years (*n* = 15)	0.82 (IQR 0.75–0.86)	1.08 (IQR 0.95–1.29)	**<0.001**	0.90 (IQR 0.86–1.06)	0.75 (IQR 0.7–0.81)	**<0.001**	1.38 (IQR 1.26–1.54)	1.04 (IQR 0.75–1.19)	**<0.001**

67 to 75 years (*n* = 17)	0.85 (IQR 0.56–0.89)	0.91 (IQR 0.80–1.28)	**0.004**	0.91 (IQR 0.84–1.23)	0.84 (IQR 0.680.97)	0.05	1.35 (IQR 1.22–2.06)	1.26 (IQR 0.78–1.38)	0.05

76 to 88 years (*n* = 12)	0.89 (IQR 0.52–1.03)	0.87 (IQR 0.77–0.91)	0.72	0.88 (IQR 0.72–1.47)	0.92 (IQR 0.901.05)	0.60	1.21 (IQR 1.08–2.14)	1.22 (IQR 1.17–1.3)	0.93

## Data Availability

The gait data used to support the findings of this study are restricted by the IRB of the Fundación Valle del Lili in order to protect patient privacy.

## References

[1] Kalia L. V., Lang A. E. (2015). Parkinson’s disease. *The Lancet*.

[2] Banta J. V. (2001). The evolution of gait analysis: a treatment decision-making tool. *Connecticut Medicine*.

[3] Viehweger E., Pfund L. Z., Hélix M. (2010). Influence of clinical and gait analysis experience on reliability of observational gait analysis (Edinburgh gait score reliability). *Annals of Physical and Rehabilitation Medicine*.

[4] Clark R. A., Vernon S., Mentiplay B. F. (2015). Instrumenting gait assessment using the Kinect in people living with stroke: reliability and association with balance tests. *Journal of NeuroEngineering Rehabilitation*.

[5] de Laat K. F., Reid A. T., Grim D. C. (2012). Cortical thickness is associated with gait disturbances in cerebral small vessel disease. *NeuroImage*.

[6] Caetano M. J. D., Lord S. R., Schoene D., Pelicioni P. H. S., Sturnieks D. L., Menant J. C. (2016). Age-related changes in gait adaptability in response to unpredictable obstacles and stepping targets. *Gait & Posture*.

[7] Judge J. O., Davis R. B., Ounpuu S. (1996). Step length reductions in advanced age: the role of ankle and hip kinetics. *Journals of Gerontology Series A: Biological Sciences and Medical Sciences*.

[8] Paker N., Bugdayci D., Goksenoglu G., Demircioğlu D. T., Kesiktas N., Ince N. (2015). Gait speed and related factors in Parkinson’s disease. *Journal of Physical Therapy Science*.

[9] Rochester L., Nieuwboer A., Baker K. (2008). Walking speed during single and dual tasks in Parkinson’s disease: which characteristics are important?. *Movement Disorders*.

[10] Nemanich S. T., Duncan R. P., Dibble L. E. (2013). Predictors of gait speeds and the relationship of gait speeds to falls in men and women with Parkinson disease. *Parkinson’s Disease*.

[11] Simon S. R. (2004). Quantification of human motion: gait analysis-benefits and limitations to its application to clinical problems. *Journal of Biomechanics*.

[12] Geerse D. J., Coolen B. H., Roerdink M. (2015). Kinematic validation of a multi-Kinect v2 instrumented 10-meter walkway for quantitative gait assessments. *PLoS One*.

[13] Clark R. A., Bower K. J., Mentiplay B. F., Paterson K., Pua Y.-H. (2013). Concurrent validity of the Microsoft Kinect for assessment of spatiotemporal gait variables. *Journal of Biomechanics*.

[14] Baker R. (2006). Gait analysis methods in rehabilitation. *Journal of NeuroEngineering and Rehabilitation*.

[15] van Diest M., Stegenga J., Wörtche H. J., Postema K., Verkerke G. J., Lamoth C. J. C. (2014). Suitability of Kinect for measuring whole body movement patterns during exergaming. *Journal of Biomechanics*.

[16] Fern’ndez-Baena A., Susin A., Lligadas X. Biomechanical validation of upper-body and lower-body joint movements of Kinect motion capture data for rehabilitation treatments.

[17] Zhao J., Bunn F. E., Perron J. M., Shen E., Allison R. S. Gait assessment using the Kinect RGB-D sensor.

[18] Clarke C. E., Patel S., Ives N. (2016). Clinical effectiveness and cost-effectiveness of physiotherapy and occupational therapy versus no therapy in mild to moderate Parkinson’s disease: a large pragmatic randomised controlled trial (PD REHAB). *Health Technology Assessment*.

[19] Arango Paredes J. D., Muñoz B., Agredo W., Ariza-Araújo Y., Orozco J. L., Navarro A. A reliability assessment software using Kinect to complement the clinical evaluation of Parkinson’s disease.

[20] Latorre J., Llorens R., Colomer C., Alcañiz M. (2018). Reliability and comparison of Kinect-based methods for estimating spatiotemporal gait parameters of healthy and post-stroke individuals. *Journal of Biomechanics*.

[21] Eltoukhy M., Kuenze C., Oh J., Jacopetti M., Wooten S., Signorile J. (2017). Microsoft Kinect can distinguish differences in over-ground gait between older persons with and without Parkinson’s disease. *Medical Engineering & Physics*.

[22] Pachoulakis I., Xilourgos N., Papadopoulos N., Analyti A. (2016). A Kinect-based physiotherapy and assessment platform for Parkinson’s disease patients. *Journal of Medical Engineering*.

[23] Ganguly B., Konar A. (2018). Kinect sensor based gesture recognition for surveillance application. http://arxiv.org/abs/1812.09595.

[24] Lun R., Zhao W. (2015). A survey of applications and human motion recognition with Microsoft Kinect. *International Journal of Pattern Recognition and Artificial Intelligence*.

[25] Mohapatra S., Swain A., Das M., Mohanty S. Real time biometric surveillance with gait recognition.

[26] Correa D. S. O., Sciotti D. F., Prado M. G., Sales D. O., Wolf D. F., Osorio F. S. Mobile robots navigation in indoor environments using Kinect sensor.

[27] Wang J. (2018). Mobile and connected health technologies for older adults aging in place. *Journal of Gerontological Nursing*.

[28] Ospina B. M., Chaparro J. A. V., Paredes J. D. A., Pino Y. J. C., Navarro A., Orozco J. L. (2018). Objective arm swing analysis in early-stage Parkinson’s disease using an RGB-D camera (Kinect®). *Journal of Parkinson’s Disease*.

[29] Dai H., Zhang P., Lueth T. (2015). Quantitative assessment of parkinsonian tremor based on an inertial measurement unit. *Sensors*.

[30] Unser M., Aldroubi A. (1996). A review of wavelets in biomedical applications. *Proceedings of the IEEE*.

[31] Ebrahimi F., Mikaeili M., Estrada E., Nazeran H. Automatic sleep stage classification based on EEG signals by using neural networks and wavelet packet coefficients.

[32] Li C., Zheng C., Tai C. (1995). Detection of ECG characteristic points using wavelet transforms. *IEEE Transactions on Biomedical Engineering*.

[33] Chau T. (2001). A review of analytical techniques for gait data. Part 2: neural network and wavelet methods. *Gait & Posture*.

[34] Cvetkovic D., Übeyli E. D., Cosic I. (2008). Wavelet transform feature extraction from human PPG, ECG, and EEG signal responses to ELF PEMF exposures: a pilot study. *Digital Signal Processing*.

[35] Akin M., Arserim M. A., Kiymik M. K., Turkoglu I. A new approach for diagnosing epilepsy by using wavelet transform and neural networks.

[36] Castano Y. J., Navarro A., Arango J. D., Muñoz B., Orozco J. L., Valderrama J. Gait and arm swing analysis measurements for patients diagnosed with Parkinson’s disease, using digital signal processing and Kinect.

[37] Roiz R. D. M., Cacho E. W. A., Pazinatto M. M., Reis J. G., Cliquet A., Barasnevicius-Quagliato E. M. A. (2010). Gait analysis comparing Parkinson’s disease with healthy elderly subjects. *Arquivos de Neuro-Psiquiatria*.

[38] Sofuwa O., Nieuwboer A., Desloovere K., Willems A.-M., Chavret F., Jonkers I. (2005). Quantitative gait analysis in Parkinson’s disease: comparison with a healthy control group. *Archives of Physical Medicine and Rehabilitation*.

[39] Baltadjieva R., Giladi N., Gruendlinger L., Peretz C., Hausdorff J. M. (2006). Marked alterations in the gait timing and rhythmicity of patients with de novo Parkinson’s disease. *European Journal of Neuroscience*.

[40] Woo J., Ho S. C., Lau J., Chan S. G., Yuen Y. K. (1995). Age-associated gait changes in the elderly: pathological or physiological?. *Neuroepidemiology*.

[41] Bendall M. J., Bassey E. J., Pearson M. B. (1989). Factors affecting walking speed of elderly people. *Age and Ageing*.

[42] Himann J. E., Cunningham D. A., Rechnitzer P. A., Paterson D. H. (1988). Age-related changes in speed of walking. *Medicine & Science in Sports & Exercise*.

[43] Herssens N., Verbecque E., Hallemans A., Vereeck L., Van Rompaey V., Saeys W. (2018). Do spatiotemporal parameters and gait variability differ across the lifespan of healthy adults? A systematic review. *Gait & Posture*.

[44] Hollman J. H., McDade E. M., Petersen R. C. (2011). Normative spatiotemporal gait parameters in older adults. *Gait & Posture*.

[45] Giladi N., Herman T., Reider-Groswasser I., Gurevich T., Hausdorff J. M. (2005). Clinical characteristics of elderly patients with a cautious gait of unknown origin. *Journal of Neurology*.

[46] Levy G. (2007). The relationship of Parkinson disease with aging. *Archives of Neurology*.

[47] Jankovic J., Kapadia A. S. (2001). Functional decline in Parkinson disease. *Archives of Neurology*.

[48] Müller B., Ilg W., Giese M. A., Ludolph N. (2017). Validation of enhanced Kinect sensor based motion capturing for gait assessment. *PLoS One*.

[49] Cunha J. P. S., Rocha A. P., Choupina H. M. P. A novel portable, low-cost Kinect-based system for motion analysis in neurological diseases.

[50] Hausdorff J. M. (2009). Gait dynamics in Parkinson’s disease: common and distinct behavior among stride length, gait variability, and fractal-like scaling. *Chaos: An Interdisciplinary Journal of Nonlinear Science*.

[51] Ko S.-U., Hausdorff J. M., Ferrucci L. (2010). Age-associated differences in the gait pattern changes of older adults during fast-speed and fatigue conditions: results from the Baltimore longitudinal study of ageing. *Age Ageing*.

